# An assessment of immediate newborn care readiness and availability in Nepal

**DOI:** 10.1080/16549716.2023.2289735

**Published:** 2023-12-12

**Authors:** Ranjan Dhungana, Mala Chalise, Robert B. Clark

**Affiliations:** a Children’s Medical Mission, Kathmandu, Nepal; b Department of Public Health, Brigham Young University, Provo, UT, USA

**Keywords:** Newborn resuscitation, immediate newborn care, health facility readiness, neonatal morbidity, newborn care capacity

## Abstract

**Background:**

Global neonatal mortality necessitates access to immediate newborn care interventions. In Nepal, disparities persist in the readiness and availability of newborn care services within health facilities.

**Objective:**

This study aimed to assess this status and compare facilities that had implemented an intensive newborn resuscitation capacity building and retention programme in the past five years with those that had not.

**Methods:**

Our observational cross-sectional study involved 154 health facilities across Nepal. Through on-site inspections and maternal log reviews, we evaluated the immediate newborn care readiness and availability.

**Results:**

The mean immediate newborn care intervention availability score of 52.8% (SE = 21.5) and the readiness score averaged 79.6% (SE = 12.3). Encouragingly, 96% of facilities ensured newborns were dried and wrapped for warmth, and 69.9% provided newborn resuscitation. Practices such as delayed cord clamping (42.0%), skin-to-skin contact (28.6%), and early breastfeeding (63.5%) showed room for improvement. Only 16.1% of health facilities administered Vitamin K1 prophylaxis.Domain-specific scores demonstrated a high level of facility readiness in infrastructure (97.5%), medicine, equipment, and supplies (90.6%), and staff training (90.9%), but a lower score for neonatal resuscitation aids (28.8%). Disparities in readiness and availability were evident, with rural areas and the Madhesh province reporting lower scores. Variations among health facility types revealed provincial and private hospitals outperforming local-level facilities. A positive association was observed between the LDSC/SSN mentoring programme and both the readiness and availability of immediate newborn care services.

**Conclusion:**

This study highlights the gap between healthcare facility readiness and the actual availability of immediate newborn care interventions in Nepal. Addressing disparities and barriers, particularly in rural areas and local-level facilities, is crucial for improving neonatal survival. The positive link between the LDSC/SSN programme and service availability and facility readiness emphasises the significance of targeted training and mentorship programmes in enhancing newborn care across Nepal.

## Introduction

Globally, remarkable progress has been achieved in reducing mortality rates under the age of five. However, an alarming trend has emerged in the Neonatal Mortality Rate (NMR), which measures the number of deaths within the first 28 days of life per 1,000 live births. The proportion of child deaths occurring in the first 28 days has exhibited a steady increase [1] and is projected to account for nearly 52% of child deaths by 2030 [2]. Tragically, the majority of neonatal deaths occur within the first week of birth and are primarily attributed to preventable medical conditions [1].

Nepal, too, has made substantial strides in child survival since 1990, achieving an impressive 72% reduction in under-five mortality rates between 1991 and 2022 [3]. However, the reduction in NMR has been slower, with only a 58% decline during the same period. The stagnation in NMR, particularly since 2016 [4], raises concerns about Nepal’s progress in ensuring neonatal health. Moreover, persistent disparities in NMR across socioeconomic groups highlight the need for targeted interventions [5].

Low-cost immediate newborn care interventions, including delayed cord clamping, thorough drying, assessment of breathing, skin-to-skin contact, early initiation of breastfeeding, and newborn resuscitation hold the potential to avert a majority of these deaths [6]. The Nepal Ministry of Health and Population (MoHP) recognises the significance of facility-based immediate newborn care strategies and has introduced several initiatives to enhance newborn survival.

These initiatives, such as the Facility-Based Integrated Management of Newborn and Childhood Illness (FB-IMNCI) [7], and the Comprehensive New-Born Care Program [8], aim to improve newborn care by training healthcare providers and ensuring the availability of essential resources. Moreover, Nepal’s alignment with Sustainable Development Goal (SDG) Target 3.2 and endorsement of the Nepal Every Newborn Action Plan-2016 (NENAP) emphasise evidence-based, cost-effective, and high-quality newborn care interventions [9]. Similarly, the Nepal Safe Motherhood and Newborn Health Road Map 2030 identify essential interventions crucial for saving newborn lives, including hypothermia management, cord clamping, newborn resuscitation, vitamin K prophylaxis, breastfeeding, and skin-to-skin care [10].

Despite these efforts, critical gaps persist in both the availability of newborn care interventions and the health facilities readiness in Nepal to deliver such care [11]. Readiness refers to the capacity of health facilities to provide immediate newborn care, assessed by factors such as presence of necessary equipment, medicines and supplies, trained staff, and adherence to guidelines [12]. The availability of immediate newborn care gauges the extent and coverage of critical interventions, including thermal management, newborn resuscitation, delayed cord cutting, and early initiation of breastfeeding, among others. Within this context, Nepal faces shortages in essential supplies, inadequacies in infrastructures, and a deficit of competent human resources capable of managing newborn complications [9]. Furthermore, access to care remains notably limited.

The COVID-19 pandemic has exacerbated the challenges of ensuring neonatal survival [13], leading to a reduction in institutional birth by half and a threefold increment in labour ward neonatal mortality during the initial two months of COVID-19-related restrictions in Nepal [14]. Research conducted across 118 Low- and Middle-Income Countries (LMICs) projected that disruptions in maternal and neonatal healthcare services caused by the pandemic could increase under-5 mortality by 9.8% to 44.7% per month, depending on the extent of disruption [15].

Given this complex landscape, it becomes imperative to conduct comprehensive assessments of health facilities, evaluate the delivery of immediate newborn care interventions, and assess the capacities of health facilities to provide these interventions at recommended quality standards. However, the existing information in this regard is limited in Nepal. To bridge these knowledge gaps, we conducted a rigorous assessment of health facilities in Nepal aimed to evaluate the status of the immediate newborn care readiness and availability at health facilities. We compared these aspects between facilities that had implemented intensive neonatal resuscitation capacity building and retention activities, utilising the Helping Babies Breathe (HBB) curriculum in the past five years with those who had received initial HBB training but no subsequent assistance. Results from this study are expected to inform evidence-based strategies to strengthen health system capacity, ensure access to quality immediate newborn care services, and support policymakers in their efforts to enhance neonatal healthcare in Nepal.

## Methods

### Study setting

Safa Sunaulo Nepal (SSN) and Latter-day Saint Charities (LDSC) in collaboration with Nepal Health Training Centre (NHTC), a division of the MoHP, implemented an extensive neonatal resuscitation capacity building, scale-up, and skill retention programme from December 2017 to November 2021 in five provinces of Nepal. This programme aimed to enhance and retain the skills and capacity of healthcare providers in immediate newborn care. The preparation included a Helping Babies Breathe (HBB) instructor training course, following the curriculum developed by the American Academy of Pediatrics. HBB is a hands-on, simulation-based training programme focused on basic newborn resuscitation for birth attendants, ensuring appropriate interventions within the first 60 seconds of life, the ‘Golden Minute’ [16]. The programme trained 443 HBB trainers and provided training materials (training resource package, neonatal resuscitation guidelines, NeoNatalie, bags and masks, and stethoscopes) for both skill practice and clinical use in 191 health facilities. In addition to this preparation for all 191 health facilities, 87 of these health facilities were provided active coaching and mentorship to facilitate facility-level scale-up and skill retention. The active coaching and mentorship averaged about 15 months per hospital during the 4 years period. Details on programme design are presented elsewhere [17].

### Study design and data collection

Ethical approval for the study was obtained from the Nepal Health Research Council with registration number 236/2022 P. This is an observational cross-sectional study to assess the immediate newborn care readiness and availability in the Nepalese health facilities. A stratified sampling strategy was used to categorise health facilities into two strata: those that received intensive mentoring support from LDSC/SSN to scale-up newborn resuscitation capacity at their facilities and those that did not. We reached out to 87 facilities that received active mentoring support from LDSC/SSN for inclusion in the study. 77 consented to participate in this study and were included in the first stratum. In the second stratum, 77 health facilities out of 104 facilities who received the initial training but did not receive the subsequent intensive mentoring support were randomly selected to be included in the study. Hence, a total of 154 health facilities were selected for inclusion in this study, with each stratum comprising 77 health facilities.

The readiness of health facilities to provide immediate newborn care was assessed through on-site inspections of facilities during which the presence and functionality of essential utilities, equipment, drugs, medical supplies, guidelines, resuscitation aids, and the presence of trained human resources as per the MoHP protocols [12,18] were determined.

The availability of immediate newborn care was assessed by reviewing maternal logs at each health facility. The secondary review of maternal logs ascertained whether immediate newborn care interventions had been provided within the three months preceding the survey and the type of interventions provided.

The readiness tool was constructed in the English language through a multi-step process. We commenced with a literature review of the MoHP protocol and other relevant literature to identify key aspects of the Nepalese health system to provide immediate newborn care, including identification of critical items and domains pertinent to our research objectives. Subject matter experts in the relevant field provided feedback on the clarity, relevance, and appropriateness of the items included in the tool. Subsequently, pilot tests were conducted in four health facilities in Banke and Sindhupalchok Districts (two each) to address issues with clarity and item wordings, leading to necessary refinements.

Data collection spanned from July to September 2022 and was led by the first author, a highly trained public health professional and HBB trainer, with an extensive background in data collection, provider training, and research at newborn health related non-government organisations. The first author led the data collection process, including protocol development, coordination with health facilities, data collection, supervision, quality control and data management. An HBB-trained medical consultant provided support in the health facilities assessment and review of maternal logs. The data collectors, led by first author, underwent workshop sessions prior to health facilities assessment to ensure consistency in data collection and interpretation. Data completeness and accuracy were maintained through regular checks by the first author. Supervisors provided oversight and quality control during the process through discussions with data collectors, and cross-checking data against the study protocol.

### Outcome variable and measurement

The study focused on two outcome variables: i) Immediate newborn care availability, and ii) Immediate newborn care readiness (Supplementary Material A).

‘Immediate newborn care availability’ was defined as the reported availability of six immediate newborn care interventions based on the National Medical Standard for Maternal and Newborn [18]:

- drying and wrapping babies to keep warm,
- newborn resuscitation,
- delayed cord clamping,
- skin-to-skin contact within the first 24 hours of life,
- initiation of breastfeeding,
- vitamin K1 prophylaxis.

The health facility was considered to have availability of immediate newborn care interventions if these services were available and utilised within the three months preceding the study.

The outcome variable ‘immediate newborn care readiness’ was defined as the preparedness and capacity of the facility to provide immediate newborn care. It was measured based on the availability and functioning of items at the time of study, grouped into four domains:

- infrastructure (6 items),
- essential medicine, equipment and supplies (13 items),
- staff and trainings (3 items),
- neonatal resuscitation aids (2 items).

These items were measured as a binary variable, considering one for both availability and functionality and 0 for unavailability or non-functionality of the item in the facility. The mean score for each domain was calculated by adding the presence of items, divided by the total number of items in the domain and multiplied by 100. 20% weightage each was given to domains infrastructure; essential medicine, equipment and supplies; and neonatal resuscitation aid, while 40% weightage was given to domain staff and trainings. The weighted average of the score from four domains formed the readiness score.

### Independent variables

The independent variables included: facility type (Provincial hospitals/Local level facilities/Private hospitals), location of the facility (rural/urban based on classification by the Government of Nepal), province (Koshi/Madhesh/Bagmati/Gandaki/Lumbini/Karnali/Sudurpaschim), and implementation of LDSC/SSN’s intensive resuscitation scale-up and retention program at the health facilities (yes/no). The hospitals under provincial government were classified as Provincial hospitals whereas the facilities (local hospital, PHCCs and HPs) under local governments were classified as Local-level facilities. Private hospitals were those owned by the private sector. Health facility classification by location was based on the type of municipality in which the health facility was located. Facilities from rural municipalities were considered rural facilities and those from a municipality, sub-metropolitan and metropolitan city were categorised as urban, in accordance with government classifications.

### Data management and statistical analysis

The survey data was collected manually and entered into Excel, followed by cleaning for accuracy, completeness and consistency. The Excel data was imported to SPSS version 26, where further statistical analysis was performed.

Descriptive analyses used the mean for continuous variables and percentages for categorical data. Mean availability of immediate newborn care was computed to analyse the status of availability of immediate newborn care at health facilities. Similarly, mean availability of each tracer item, domain-specific readiness score, and overall immediate newborn care readiness score were calculated in the analysis of health facility readiness to provide immediate newborn care. Pearson’s Chi-Square test and Kruskal-Wallis test were used to assess association of independent variables with the mean availability and readiness score, respectively. A *p* value of less than 0.05 was considered a statistically significant association.

## Results

### General characteristics of health facilities

Table 1 presents the characteristics of the facilities included in the study. Of the total facilities, the majority were from rural area (63.1%) and were Local-level facilities (74.4%). Lumbini province accounted for 31.4% of the facilities. Nearly 37% of the facilities received LDSC/SSN’s one-two year newborn resuscitation capacity building and skill retention programme. A detailed description of the health facilities, including the nature and scope of services they provide, is given in Supplementary Material B.

**Table 1.** General characteristics of health facilities (*n* = 154).

Background Characteristics	Unweighted frequency (%)	Weighted frequency (%)
**Facility Location**		
Rural	69 (44.9)	133 (63.1)
Urban	85 (55.1)	78 (36.9)
**Facility Type**		
Local level facilities	79 (51.3)	157 (74.4)
Provincial level Hospitals	49 (31.8)	3 (1.4)
Private Hospitals	26 (16.9)	51 (24.2)
**Province**		
Koshi	16 (10.4)	24 (11.3)
Madhesh	34 (22.1)	50 (23.8)
Bagmati	16 (10.4)	20 (9.5)
Gandaki	20 (13.0)	20 (9.7)
Lumbini	45 (29.2)	66 (31.4)
Karnali	16 (10.4)	20 (9.6)
Sudurpashchim	7 (4.5)	10 (4.8)
**Implementation of LDSC/SSN’s newborn resuscitation capacity building and skill retention program**		
Yes	77 (50.0)	78 (36.8)
No	77 (50.0)	134 (63.2)

### Immediate newborn care availability

**Figure 1.**
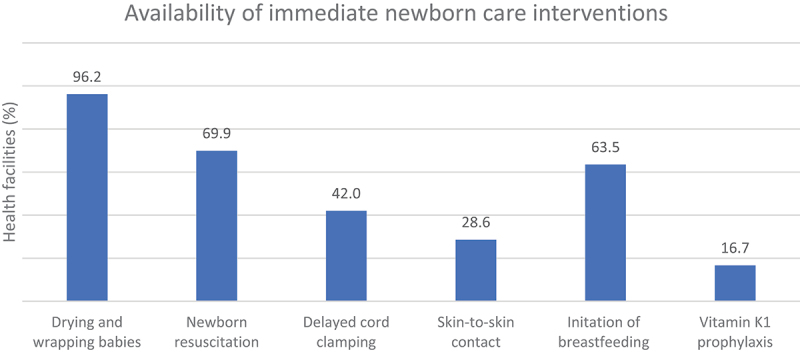
Availability of immediate newborn care interventions in health facilities.

The mean immediate newborn care intervention availability score was 52.8% (SE = 21.50) (Table 2). The mean availability of immediate newborn care interventions was higher among facilities in urban areas (57.2%, SE = 1.98) compared to rural areas (50.3%, SE- 2.022, *p* = 0.029). Provincial-level facilities had a higher mean availability score (61.6%, SE = 11.89) than other facility types. Similarly, immediate newborn care was more available in Karnali province (75%, SE = 3.51, *p *= <0.001) rather than in other provinces. Likewise, availability scores were higher in health facilities receiving implementation of LDSC/SSN’s newborn resuscitation capacity building and skill retention programme (70%, SE = 1.71, *p *= <0.001) compared to non-receiving facilities. The distribution of the six immediate newborn care interventions is presented in Figure 1. Approximately 96% of the facilities dried and wrapped babies to keep them warm, 69.9% provided newborn resuscitation, 42.0% provided delayed cord clamping, 28.6% of health facilities kept skin-to-skin contact with mother and 63.5% of the health facilities initiated early breastfeeding. Vitamin K1 prophylaxis was administered to the newborns in only 16.1% of health facilities. The distribution of each element of immediate newborn care availability by background characteristics is provided in the Supplementary Material C.

**Table 2.** Immediate newborn care availability and readiness score by background characteristics.

Background Characteristics	Weighted Mean Availability Score %, (SE)	*p*-value^a^	Weighted Mean Readiness Score %, (SE)	*p*-value^a^
**Overall**	52.8, (21.50)	–	79.6, (12.27)	–
**Facility Location**				
Rural	50.3, (2.02)	**0.029**	76.1, (0.98)	**<0.001**
Urban	57.2, (1.98)		85.5, (1.32)	
**Facility Type**				
Local level facilities	53.8, (1.89)	0.183	77.4, (0.93)	**<0.001**
Provincial level Hospitals	61.6, (11.89)		92.4, (7.12)	
Private Hospitals	49.3, (1.78)		85.5, (1.69)	
**Province**				
Koshi	52.3, (3.70)	**<0.001**	77.7, (1.34)	**<0.007**
Madhesh	35.5, (2.95)		76.5, (2.03)	
Bagmati	63.6, (4.09)		83.1, (2.40)	
Gandaki	55.1, (4.77)		84.1, (2.70)	
Lumbini	53.6, (2.02)		77.4, (1.58)	
Karnali	75, (3.51)		87.1, (1.42)	
Sudurpashchim	63.3, (4.14)		82.4, (3.29)	
**Implementation of LDSC/SSN’s newborn resuscitation capacity building and skill retention program**				
Yes	70.0, (1.71)	**<0.001**	91.2, (0.96)	**<0.001**
No	42.8, (1.57)		72.8, (0.73)	

a= Kruskal-Wallis test, *significant value <0.05.

### Health facility readiness for immediate newborn care

The mean facility readiness score to provide immediate newborn care was 79.6% (SE = 12.27) (Table 2). The four-domain mean readiness score was higher among facilities in urban areas (85.5%, SE = 1.32)

compared to rural areas (76.1%, SE = 0.98, *p* < 0.001). Provincial-level facilities had a higher mean readiness score (92.4%, SE = 7.13, *p* < 0.001). Similarly, Karnali province had the highest mean readiness score (87.1%, SE = 1.42) while Madhesh province had the lowest mean readiness score (76.5%, SE = 2.03, *p* = 0.01). Likewise, the readiness score was higher in health facilities that received LDSC/SSN’s newborn resuscitation capacity building and skill retention programme (91.2%, SE = 0.96) compared to non-receiving facilities (72.8%, SE = 0.73, *p* < 0.001).

**Figure 2.**
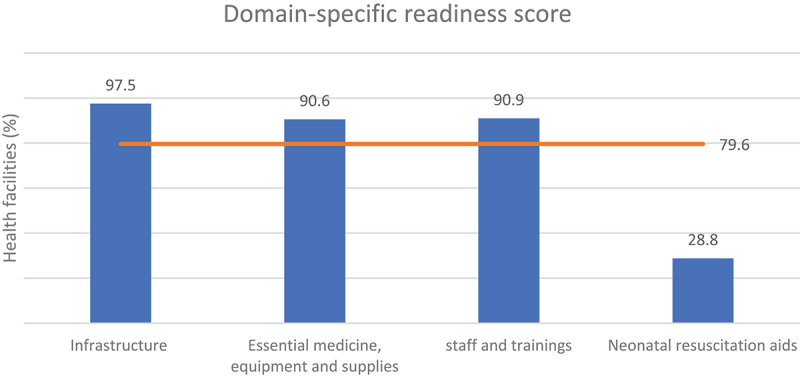
Domain-specific readiness score for immediate newborn care in health facilities.

The domain-specific readiness score for infrastructure; medicine, equipment and supplies; staff and trainings; and neonatal resuscitation aids were 97.5%, 90.6%, 90.9%, and 28.8% respectively (Figure 2). The domain-specific readiness score by background characteristics is presented in the Supplementary Material D.

The majority of the tracer items under domain infrastructure; and essential medicine, equipment and supplies; and staff and trainings were available in the health facilities. Availability of vitamin K1 injection was considerably low at 18.6% of health facilities (Supplementary Material E).

## Discussion

This study assessed the availability and readiness of health facilities to provide immediate newborn care interventions in Nepal. Our study stands out among other health facilities assessments in Nepal because of its specialised focus on immediate newborn care interventions. While recent surveys such as the Nepal Health Facility Survey (NHFS) [19] provide essential insights into healthcare system readiness to provide a broad array of health services, our study delves deeper into the critical area of newborn care during the crucial moments following birth.

We assessed the readiness and availability of health facilities to provide life-saving interventions for newborns, shedding light on vital aspects such as the administration of vitamin K1, skin-to-skin contact, delayed cord clamping, neonatal resuscitation, early initiation of breastfeeding and availability of infrastructures to support the delivery of these interventions. Notably, our study addresses the presence of newborn resuscitation practice aids, a pivotal yet often overlooked component of newborn care. By emphasising on these specific interventions, our study offers a nuanced and policy-relevant perspective on neonatal care, providing targeted recommendations to enhance the quality of care for newborns in Nepal’s healthcare system.

The study revealed a significant gap between the capacity of health facilities to deliver immediate newborn care and actual availability of such care at health facilities. While healthcare facilities demonstrated reasonable readiness, the study found that the actual coverage of immediate newborn care interventions fell notably short. This gap underscores the need to address underlying barriers impeding the delivery of recommended newborn care interventions within these facilities.

Disparities in readiness and availability were evident across locations, with urban areas and specific provinces demonstrating higher scores for both readiness and availability. Conversely, rural areas and the Madhesh province exhibited lower scores, underscoring the fact that a substantial proportion of neonatal deaths occur in these regions [5]. Additionally, disparities were observed among different types of health facilities, with Provincial and private hospitals outperforming local level facilities. Given that a significant number of deliveries take place in Local-level facilities [10], enhancing their capacity for immediate newborn care holds substantial potential for improving neonatal survival.

The analysis highlighted a positive association between the LDSC/SSN’s programme and both the readiness and availability of immediate newborn care services (Supplementary Material C and D). The programme’s focus on training and skill retention among birth attendants appears to have not only positively influenced the delivery of essential newborn care interventions, but also influenced the readiness of facilities to care for newborns. This correlation between maintaining provider’s skills and facility readiness may be the result of increased attention to newborn outcomes and has potential policy implications.

Specific interventions, such as vitamin K1 administration after the first hour of birth, skin-to-skin contact and delayed cord cutting, faced challenges. Vitamin K1 administration was notably low, with only 18.6% of the health facilities possessing the injection and 16.1% of the health facilities providing it, reflecting a broader issue of logistical challenges and policy integration [20]. This finding aligns with the results of a recent nationally representative survey of health facilities in Nepal, which also reported a similar lower availability of vitamin K1 at health facilities [19].

Similarly, the coverage of skin-to-skin contact remained relatively low, despite government prioritisation of this intervention [21]. The finding is consistent with an observational study of four referral level public hospitals in Nepal which found that skin-to-skin contact was practiced in only 3.50% of babies who breathed spontaneously after birth [22]. The lack of trained and motivated healthcare staff, along with logistical challenges affects its availability [23].

As with a previous study [24], gaps were observed in delayed cord cutting, with notable disparities among different types of health facilities (Supplementary Material C). This may be due to the lack of national and institutional protocols and inadequate training support for delivery staff [25]. These findings emphasise the need for targeted efforts to improve the availability of these critical interventions.

Neonatal resuscitation coverage, however, exceeded national data (69.9% vs. 29.6%) [18], and reflects the fact that all facilities were exposed to HBB training, with one-half of the facilities receiving intensive retention efforts. It is also of note that many of the facilities had received HBB training 3–5 years prior to this survey. Thus, HBB may have improved the proficiency of healthcare providers in identifying non-breathing newborns at birth and providing newborn resuscitation in the facilities assessed even when training was not current.

In terms of readiness domains, healthcare facilities generally exhibited reasonable availability of essential infrastructure, equipment, medicines, supplies, and human resources, suggesting compliance with MoHP standards. Our findings on newborn corner availability (Supplementary Material E) specifically contrast with the NHFS data, which reports lower availability (44.6%) at facilities [19]. All health facilities included in the study were provided with minimal supplies to establish and maintain newborn corners by LDSC/SSN at the conclusion of the TOT. Consequently, this support likely contributed to the establishment and sustained functionality of newborn corners, leading to the observed increased availability in our study.

However, the domain of neonatal resuscitation aids, which assessed the presence of newborn resuscitation management guidelines and a practice NeoNatalie in healthcare facilities, received the lowest score. These neonatal resuscitation aids play a critical role in preparing and supporting healthcare providers to care for non-breathing infants, reflecting their past skills training, current preparation, and capacity for performing resuscitation [26]. LDSC/SSN supplied NeoNatalie practice manikins and guideline posters to all the healthcare facilities included in the study following the HBB TOT. Nevertheless, the limited availability of neonatal resuscitation aids underscores a significant challenge that must be addressed to ensure the delivery of comprehensive immediate newborn care services in healthcare facilities. Facilities that received mentoring support from LDSC/SSN demonstrated significantly higher availability of these aids (Supplementary Material D), despite the varying time lag between support and survey. This highlights the positive impact of mentorship on promoting frequent skills practice and emphasising the importance of utilising NeoNatalie practice to adhere to newborn resuscitation guidelines.

To achieve Nepal’s SDG goal of reducing the neonatal mortality rate, it is imperative that the government focuses on enhancing access to critical interventions, like vitamin K1 administration, promoting skin-to-skin contact, and encouraging delayed cord clamping. Mentorship programmes, akin to the LDSC/SSN’s model or a dedicated clinical leadership, and implementation of targeted interventions to address inequities in preparedness and availability of immediate newborn care could be a reasonable approach to tackle these issues.

The limitations of our study include the use of point prevalence during the observation to assess availability and readiness in providing newborn care and the absence of determining round-the-year availability of six immediate newborn care interventions and related tracer items. Moreover, it is possible that the use of purposive sampling might have led to selection bias for geographically accessible facilities. Additionally, there might be potential variation in neonatal mortality rates among the health facilities, thereby limiting the generalisability of the findings to a broader population. Lastly, this study solely assessed the infrastructural aspect of health facilities to provide six immediate newborn care interventions, and it does not guarantee the presence of necessary skills and processes to provide high-quality care for newborns.

## Conclusion

This study underscores the gap between healthcare facility readiness and the actual availability of immediate newborn care interventions in Nepal. Addressing disparities and barriers, particularly in rural areas and Local level facilities, is crucial for improving neonatal survival. The positive association between the LDSC/SSN programme and both service availability and infrastructure readiness highlights the importance of targeted training and mentorship programmes. Further research and policy efforts are needed to enhance the coverage of specific interventions and improve overall newborn care quality.

## Supplementary Material

supplementary Material C.docx

Supplementary Material B.docx

Supplementary Material E.docx

supplementary Material D.docx

Supplementary Material A.docx

## Paper context

Global neonatal mortality highlights the importance of standard newborn interventions. We found varying availability of key interventions and facility infrastructure readiness with disparities across locations, provinces, facility types, and facilities with resuscitation skill retention programmes. Disparities existed in the availability of the key interventions of delayed cord clamping, skin-to-skin contact, and administration of Vitamin K1. Addressing disparities in availability and readiness to deliver immediate newborn care is critical for enhancing neonatal survival rates.
