# Supplementary material for: An assessment of immediate newborn care readiness and availability in Nepal
Source: Glob Health Action. 2023 Dec 12;16(1):2289735. doi: 10.1080/16549716.2023.2289735 (PMC10795551; doi:10.1080/16549716.2023.2289735)
Supplement: supplementary Material C.docx [file ZGHA_A_2289735_SM8923.docx]

**Supplementary Material C. Availability of Immediate Newborn Care by background characteristics.**

| **Background Characteristics** | **Drying and wrapping n(%)** | **p-value^a^** | **Newborn resuscitation (%)** | **p-value^a^** | **Delayed Cord Clamping (%)** | **p-value^a^** | **Skin-to-skin contact (%)** | **p-value^a^** | **Initiation of breastfeeding (%)** | **p-value^a^** | **Vitamin K1 prophylaxis (%)** | **p-value^a^** |
| --- | --- | --- | --- | --- | --- | --- | --- | --- | --- | --- | --- | --- |
| **Facility Location** | | | | | | | | | | | | |
| **Rural** | 127 (95.5%) | 0.713 | 76 (56.7%) | **<0.001** | 60 (45.1%) | 0.312 | 40 (30.1%) | 0.753 | 98 (73.1%) | **<0.001** | 2 (1.5%) | **<0.001** |
| **Urban** | 76 (97.4%) |  | 72 (92.3%) |  | 29 (37.2%) |  | 21 (26.9%) |  | 37 (47.4%) |  | 33 (42.3%) |  |
| **Facility Type** | | | | | | | | | | | | |
| **Local level facilities** | 151 (96.2%) | 0.941 | 98 (62%) | **<0.001** | 80 (50.6%) | **<0.001** | 54 (34.4%) | **0.008** | 123 (78.3%) | **<0.001** | 2 (1.3%) | **<0.001** |
| **Provincial level Hospitals** | 3 (100%) |  | 3 (100%) |  | 1 (33.3%) |  | 1 (33.3%) |  | 1 (33.3%) |  | 2 (66.7%) |  |
| **Private Hospitals** | 49 (96.1%) |  | 47 (92.2%) |  | 8 (15.7%) |  | 6 (11.8%) |  | 10 (19.6%) |  | 32 (61.5%) |  |
| **Province** | | | | | | | | | | | | |
| **Koshi** | 24 (100%) | **<0.001** | 22 (91.7%) | **<0.001** | 4 (16.7%) | **<0.001** | 8 (33.3%) | **<0.001** | 6 (25%) | **<0.001** | 12 (50%) | **<0.001** |
| **Madhesh** | 42 (84%) |  | 26 (52%) |  | 8 (16%) |  | 4 (8%) |  | 22 (44%) |  | 4 (8%) |  |
| **Bagmati** | 20 (100%) |  | 18 (90%) |  | 12 (60%) |  | 8 (40%) |  | 14 (70%) |  | 4 (20%) |  |
| **Gandaki** | 20 (100%) |  | 12 (60%) |  | 10 (50%) |  | 8 (40%) |  | 12 (60%) |  | 4 (20%) |  |
| **Lumbini** | 66 (100%) |  | 40 (60%) |  | 32 (48.5%) |  | 12 (18.2%) |  | 52 (78.8%) |  | 10 (15.2%) |  |
| **Karnali** | 20 (100%) |  | 20 (100%) |  | 16 (80%) |  | 16 (80%) |  | 18 (90%) |  | 0 (0%) |  |
| **Sudurpashchim** | 10 (100%) |  | 8 (80%) |  | 6 (60%) |  | 4 (40%) |  | 10 (100%) |  | 0 (0%) |  |
| **Implementation of LDSC/SSN’s newborn resuscitation capacity building and skill retention program** | | | | | | | | | | | | |
| **Yes** | 78 (100%) | **0.028** | 78 (100%) | **<0.001** | 47 (60.3%) | **<0.001** | 46 (59.7%) | **<0.001** | 57 (73.1%) | **0.038** | 21 (26.9%) | **0.004** |
| **No** | 126 (94%) |  | 70 (52.2%) |  | 42 (31.3%) |  | 14 (10.4%) |  | 78 (58.2%) |  | 14 (10.5%) |  |

*a= Pearson Chi-Square test, *significant value <0.05*
