# Supplementary material for: An assessment of immediate newborn care readiness and availability in Nepal
Source: Glob Health Action. 2023 Dec 12;16(1):2289735. doi: 10.1080/16549716.2023.2289735 (PMC10795551; doi:10.1080/16549716.2023.2289735)
Supplement: Supplementary Material B.docx [file ZGHA_A_2289735_SM8922.docx]

**Supplementary Material B. Description of types and scope of health facilities included in the study.**

The Nepalese health system is organized into three tiers - Federal, Provincial and Local - in line with governance system as per the new constitution in 2015 ^1^. Federal hospitals offer tertiary and super-specialty maternal and newborn care as these hospitals have advanced infrastructures and staff for providing maternal and newborn care, including higher bed capacity, Neonatal Intensive Care Units (NICUs), and pediatricians. Provincial hospitals provide Comprehensive Emergency Obstetrics and Newborn Care (CEmONC). Local-level health facilities are community level health facilities such as Municipal hospitals, Primary Health Care Centers (PHCCs) and Health Posts. Municipal Hospitals and PHCCs are Basic Emergency Obstetrics and Newborn Care (BEmONC) sites and serve as the first referral sites. Some HPs are designated as Birthing Centers and provide normal delivery services ^2^. Private hospitals generally provide maternal and neonatal services in line with tertiary hospitals.

References

1.         Government of Nepal. *The Constitution of Nepal*.; 2015. Accessed June 12, 2022. <https://www.wipo.int/edocs/lexdocs/laws/en/np/np029en.pdf>.

2.         Government of Nepal. *Nepal Health Infrastructure Development Standards 2017*. Ministry of Health Accessed May 10, 2022. <https://dohs.gov.np/wp-content/uploads/2019/02/Health-Infrastructure-Development-Standards-2074-BS-Unofficial-Translation-Volume-4.pdf>
