# Supplementary material for: An assessment of immediate newborn care readiness and availability in Nepal
Source: Glob Health Action. 2023 Dec 12;16(1):2289735. doi: 10.1080/16549716.2023.2289735 (PMC10795551; doi:10.1080/16549716.2023.2289735)
Supplement: Supplementary Material E.docx [file ZGHA_A_2289735_SM8921.docx]

**Supplementary Material E. Availability of tracer items.**
