# Supplementary material for: An assessment of immediate newborn care readiness and availability in Nepal
Source: Glob Health Action. 2023 Dec 12;16(1):2289735. doi: 10.1080/16549716.2023.2289735 (PMC10795551; doi:10.1080/16549716.2023.2289735)
Supplement: supplementary Material D.docx [file ZGHA_A_2289735_SM8920.docx]

**Supplementary Material D. Domain-specific readiness score by background characteristics.**

| **Background Characteristics** | **Domain-specific readiness score (%)** | | | | | | | |
| --- | --- | --- | --- | --- | --- | --- | --- | --- |
|  | **Infrastructure** | **p-value**^a^ | **Medicine, equipment and supplies** | **p-value**^a^ | **Staff and trainings** | **p-value**^a^ | **Neonatal resuscitation aids** | **p-value** |
| **Overall** | 97.48% | - | 90.55% | - | 90.94% | - | 28.80% | **-** |
| **Facility Location** | | | | | | | | |
| Rural | 96.62 | **0.003** | 89.43 | **0.001** | 88.56 | 0.58 | 17.22 | **<0.001** |
| Urban | 99.13 |  | 92.45 |  | 93.80 |  | 48.55 |  |
| **Facility Type** | | | | | | | | |
| Local level facilities | 96.84 | **0.009** | 89.29 | **<0.001** | 89.03 | **0.029** | 22.78 | **0.004** |
| Provincial level Hospitals | 99.32 |  | 96.23 |  | 92.52 |  | 81.63 |  |
| Private Hospitals | 99.36 |  | 94.08 |  | 94.87 |  | 44.23 |  |
| **Province** | | | | | | | | |
| Koshi | 97.23 | **<0.001** | 88.55 | **0.002** | 97.18 | **0.005** | 8.47 | **0.002** |
| Madhesh | 99.32 |  | 91.72 |  | 85.39 |  | 20.75 |  |
| Bagmati | 100 |  | 93.90 |  | 90.13 |  | 41.07 |  |
| Gandaki | 96.71 |  | 91.64 |  | 92.92 |  | 46.11 |  |
| Lumbini | 96 |  | 89.12 |  | 86.96 |  | 27.74 |  |
| Karnali | 100 |  | 90.10 |  | 100 |  | 45.58 |  |
| Sudurpashchim | 90.13 |  | 90.83 |  | 100 |  | 30.90 |  |
| **Implementation of LDSC/SSN’s newborn resuscitation capacity building and skill retention program** | | | | | | | | |
| No | 96.02 | **<0.001** | 89.32 | **<0.001** | 88.58 | **0.017** | 2.32 | **<0.001** |
| Yes | 99.99 |  | 94.38 |  | 93.79 |  | 74.29 |  |

*a= Kruskal-Wallis test, *significant value <0.05*
