# Supplementary material for: An assessment of immediate newborn care readiness and availability in Nepal
Source: Glob Health Action. 2023 Dec 12;16(1):2289735. doi: 10.1080/16549716.2023.2289735 (PMC10795551; doi:10.1080/16549716.2023.2289735)
Supplement: Supplementary Material A.docx [file ZGHA_A_2289735_SM8919.docx]

**Supplementary Material A. Immediate newborn care intervention and standards.**

| **Availability of Immediate newborn care** | |
| --- | --- |
| **Intervention** | **Standard** |
| Drying and wrapping babies to keep warm | All babies are dried immediately after birth. |
| Newborn Resuscitation | In neonates who do not start breathing after thorough drying and rubbing the back 2–3 times, suctioning of mouth and nose should be done only if the mouth or nose is full of secretions or meconium before initiating positive pressure ventilation |
| Delayed Cord clamping | Cord clamping is done after 1 to 3 minutes of birth (delayed cord clamping) in all normal newborns who cry immediately. |
| Skin-to-skin contact within the first hour of life | All newborns without complications should be kept in skin-to-skin contact with their mothers during the first hour after birth to prevent hypothermia and promote breastfeeding |
| Initiation of breastfeeding | All newborns should be put to the breast as soon as possible after birth when they are clinically stable, and the mother and baby are ready |
| Vitamin K1 prophylaxis | All newborns should be given 1 mg of vitamin K1 IM (for less than 1.0 kg, 0.5 mg) after first hour of birth during which the infant should be in skinto-skin contact with the mother and breastfeeding should be initiated |
| **Health facility readiness for immediate newborn care** | |
| **Domains** | **Items** |
| Infrastructure | Power supply, labor room, handwashing area in labor room, clean utility room, newborn care corner, a sterilizer or an autoclave |
| Essential medicine, equipment and supplies | Vitamin K1, Gentamycin, Amoxicillin, Stethoscope, Infant Weighing Scale, Thermometer, Clock with second hand, Neonatal resuscitation B&M (mask size 0 and 1), Mucus extractor, Disposable Syringe, Baby blanket, Disposable gloves, Cord clam/ties, |
| Staff and trainings | midwife present at the health facility or on-call twenty-four hours to provide delivery service, at least one staff trained on Helping Babies Breathe (HBB) training in past 24 months, at least one staff trained on Skilled Birth Attendant (SBA) training in past 24 months |
| Neonatal Resuscitation Aids | Newborn Resuscitation Management Guidelines, NeoNatalie |
